# Cardiovascular risk assessment of dyslipidemic middle-aged adults without overt cardiovascular disease over the period of 2009–2016 in Lithuania

**DOI:** 10.1186/s12944-018-0883-5

**Published:** 2018-10-11

**Authors:** Sandra Kutkiene, Zaneta Petrulioniene, Aleksandras Laucevicius, Pranas Serpytis, Vytautas Kasiulevicius, Justina Staigyte, Akvile Saulyte, Emilija Petrulionyte, Urte Gargalskaite, Egle Skiauteryte, Gabija Matuzeviciene, Milda Kovaite, Egidija Rinkuniene

**Affiliations:** 1 Vilnius University, Faculty of Medicine, Clinic of Cardiac and Vascular Diseases, Vilnius, Lithuania; 2 Vilnius University, Faculty of Medicine, Clinic of Internal Diseases, Family Medicine and Oncology, Vilnius, Lithuania; 3 Vilnius University, Faculty of Medicine, Vilnius, Lithuania; 4 Vilnius University Hospital Santaros Klinikos, Santariskiu str. 2, 08661 Vilnius, Lithuania

**Keywords:** Dyslipidemia, Cardiovascular risk, Primary prevention, Clustering of risk factors, Middle-aged population, SCORE

## Abstract

**Background:**

Cardiovascular mortality in Lithuania is extremely high and abnormal lipid levels are very common among Lithuanian adults. Dyslipidemia is one of the main independent risk factors for cardiovascular diseases (CVD) leading to high absolute CVD risk. The aim of this study was to assess CVD risk in dyslipidemic middle-aged subjects.

**Methods:**

During the period of 2009–2016 a total of 92,373 people (58.4% women and 41.6% men) were evaluated. This study included men aged 40–54 and women aged 50–64 without overt CVD.

**Results:**

Any type of dyslipidemia was present in 89.7% of all study population. 7.5% of dyslipidemic patients did not have any other conventional risk factors. Three and more risk factors were detected in 60.1% of dyslipidemic subjects. All analyzed risk factors, except smoking, were more common in dyslipidemic adults compared to subjects without dyslipidemia: arterial hypertension (55.8% vs. 43.3%, *p* < 0.001), diabetes (11.1% vs. 7.3%, *p* < 0.001), abdominal obesity (45.3% vs. 30.2%, *p* < 0.001), BMI ≥30 kg/m^2^ (35.8% vs. 23.7%, *p* < 0.001), metabolic syndrome (34.0% vs. 9.2%, *p* < 0.001), family history of coronary heart disease (26.3% vs. 23.1%, *p* < 0.001), unbalanced diet (62.5% vs. 52.9%, *p* < 0.001) and insufficient physical activity (52.0% vs. 44.2%, *p* < 0.001). The prevalence of all evaluated risk factors, except smoking, increased with age. Average SCORE index was 1.87 in all study population, while dyslipidemic subjects had higher SCORE compared to control group (1.95 vs 1.20, *p* < 0.001).

**Conclusions:**

Almost two thirds of dyslipidemic middle-aged Lithuanian adults without overt cardiovascular disease had three or more other CVD risk factors, which synergistically increase absolute risk of CVD. The average 10-year risk of CVD death in patients with dyslipidemia was 1.95%. The importance of managing dyslipidemia as well as other risk factors in order to reduce burden of cardiovascular disease in Lithuania is evident.

## Background

Cardiovascular disease (CVD) is a major cause of premature death in Lithuania as more than a half of all deaths (56.2%) were caused by CVD in 2016 [1]. Our country has the highest level of deaths from coronary heart disease (CHD) in Europe [2] and is classified as high-risk country in 2016 European guidelines on CVD prevention [3]. The main risk factors for CVD are elevated levels of blood lipids, high blood pressure, tobacco use, diabetes mellitus, unhealthy eating habits, low physical activity, overweight and obesity [4].

Dyslipidemia is one of the most important modifiable risk factors described more than half a century ago [5]. The association between increased lipid concentrations and the risk of CVD is well established [6] and the causal relationship is supported by strong epidemiological evidence of efficacy of lipid lowering therapy in reducing the incidence of CHD [7]. Dyslipidemia is often found together with multiple other cardiovascular risk factors, especially hypertension and obesity [8]. It is well known that coexisting multiple risk factors tend to increase the CVD risk synergistically because of additional adverse effect on the vascular endothelium [9, 10]. Estimated prevalence of dyslipidemia in Lithuania is very high (88.8%) according to Lithuanian High Cardiovascular Risk programme data analysis [11].

It is settled that prevention is effective in reducing the impact of CVD and one of the main measures is lowering increased levels of modifiable cardiovascular risk factors [12]. Despite positive changes in prevalence of adult arterial hypertension (AH) in Lithuania, the cardiovascular risk of the population still remains high [13, 14]. While continuing the positive trends, nowadays all the efforts should be focused on management of dyslipidemia. The aim of our study was to assess cardiovascular risk profile of middle-aged Lithuanian adults with and without dyslipidemia in order to broadly analyze the importance of this major CVD risk factor.

## Methods

### Study population and measurements

The Lithuanian High Cardiovascular Risk (LitHiR) primary prevention programme, funded by the Ministry of Health, was started in 2006. The Local Research Ethics Committee’s approval was obtained. During the period of 2009–2016 a total of 92,373 people (58.4% women and 41.6% men) were evaluated. This study included men aged 40–54 and women aged 50–64 without overt cardiovascular disease. Subjects were divided into two groups by their dyslipidemia status – dyslipidemic and non-dyslipidemic. Their risk factors and lipid profile assessment were obtained and used for statistical analysis. Study data has been further analyzed by dividing all subjects into appropriate groups by the age, men: 40–44 years, 45–49 years, 50–54 years and women: 50–54 years, 55–59 years, 60–64 years.

Dyslipidemia was considered if serum total cholesterol (TC) > 5 mmol/L, or low-density lipoprotein-cholesterol (LDL-C) > 3 mmol/L, or high-density lipoprotein cholesterol (HDL-C) < 1.0 mmol/L in men and < 1.2 mmol/L in women, or triglycerides (TG) > 1.7 mmol/L. Metabolic syndrome (MS) was assessed according to the National Cholesterol Education Program III modified criteria [15]. Arterial hypertension (AH) was defined as systolic blood pressure ≥ 140 mmHg and/or diastolic blood pressure ≥ 90 mmHg, or the diagnosis of hypertension was documented in a medical record. Obesity was identified whenever body mass index (BMI) ≥30 and abdominal obesity was determined when waist circumference was > 102 cm for men and > 88 cm for women. The overall cardiovascular risk was calculated according to the risk estimation Systematic Coronary Risk Evaluation (SCORE) system [16]. A detailed description of the Lithuanian High Cardiovascular Risk programme protocol is presented in Laucevicius et al. paper [17].

### Statistical analysis

For continuous variables, the following descriptive statistics are reported: means, standard deviations (SD) and 95% confidence interval (CI). For categorical data frequencies are reported. In the case of dichotomous categorical variables, we also provide confidence intervals for proportions of interest (e.g. diabetes, smoking, etc.). These intervals were obtained using the relationship between beta and binomial distributions. Categorical variables were compared with the help of the chi-square test. All reported *p*-values are two-tailed. The level of significance was set to 0.05.

## Results

### Sample characteristics and cardiovascular risk profile of subjects with and without dyslipidemia

This study included 92,373 adults without overt cardiovascular disease – 53,961 (58.4%) women and 38,412 (41.6%) men. The average age of the sample group was 52.15 (±6.21) years. Women and men varied in age because of different study enrollment criteria. Baseline characteristics and prevalence of cardiovascular risk factors of the whole study population are shown in Table 1. 81.7% of subjects had TC > 5 mmol/L, 79.3% had LDL-C > 3 mmol/L, 30.4% had TG > 1.7 mmol/L and 13.7% had low HDL-C (< 1.0 mmol/L in men and < 1.2 mmol/L in women). Any type of dyslipidemia was diagnosed in 89.7% of middle-aged adults without overt cardiovascular disease. Group with dyslipidemia consists of 82,893 (89.7%) subjects and group without dyslipidemia includes 9480 (10.3%) adults. All the major risk factors, including AH, abdominal obesity, MS, DM and obesity, except for smoking, were more prevalent in patients with dyslipidemia compared to patients without it (*p* < 0.001) (Table 1). Average SCORE index of the whole study population was 1.87, patients with dyslipidemia had higher SCORE compared to control group (Table 1).

**Table 1:** Baseline characteristics and trends of cardiovascular risk factors in study population

	Total (*n* = 92,373)		Patients with dyslipidemia (*n* = 82,893)		Patients without dyslipidemia (*n* = 9480)		*p*-value
	Mean	SD	Mean	SD	Mean	SD	
Age (years)	52.15	6.21	52.34	6.20	50.54	6.05	< 0.001
Waist circumference (cm)	93.72	13.52	94.07	13.53	90.64	13.08	< 0.001
BMI (kg/m^2^)	28.60	5.41	28.78	5.39	27.04	5.38	< 0.001
SBP (mmHg)	133.52	16.33	133.87	16.39	130.43	15.44	< 0.001
DBP (mmHg)	82.76	9.48	82.95	9.50	81.15	9.14	< 0.001
HR (beats/min.)	71.95	8.78	72.00	8.78	71.50	8.72	< 0.001
Fasting glucose (mmol/l)	5.52	1.22	5.54	1.23	5.35	1.08	< 0.001
TC (mmol/l)	6.08	1.21	6.28	1.12	4.40	0.45	< 0.001
LDL-C (mmol/l)	3.87	1.08	4.04	1.00	2.42	0.43	< 0.001
HDL-C (mmol/l)	1.54	0.46	1.54	0.47	1.58	0.37	< 0.001
TG (mmol/l)	1.59	1.16	1.66	1.19	0.93	0.31	< 0.001
Non-HDL-C	4.54	1.21	4.74	1.11	2.83	0.48	< 0.001
TG /HDL	1.22	1.55	1.29	1.62	0.63	0.28	< 0.001
SCORE index	1.87	1.68	1.95	1.71	1.20	1.18	< 0.001
Frequencies	n	%	N	%	n	%	
DM (%)	9897	10.7%	9207	11.1%	690	7.3%	< 0.001
AH (%)	50,317	54.5%	46,216	55.8%	4101	43.3%	< 0.001
Abdominal obesity (%)	40,408	43.7%	37,547	45.3%	2861	30.2%	< 0.001
Smoking (%)	21,218	23.0%	18,703	22.6%	2515	26.5%	< 0.001
MS (%)	29,094	31.5%	28,219	34.0%	875	9.2%	< 0.001
RF ≥ 3 (%)	53,971	58.4%	49,819	60.1%	4152	43.8%	< 0.001
Familial CHD (%)	24,025	26.0%	21,837	26.3%	2188	23.1%	< 0.001
Unbalanced diet (%)	56,800	61.5%	51,783	62.5%	5017	52.9%	< 0.001
Insufficient physical activity (%)	47,268	51.2%	43,074	52.0%	4194	44.2%	< 0.001
BMI < 25 (kg/m^2^) (%)	24,891	26.9%	21,037	25.4%	3854	40.7%	< 0.001
BMI 25–30 (kg/m^2^) (%)	35,589	38.5%	32,209	38.9%	3380	35.7%	< 0.001
BMI 30–40 (kg/m^2^) (%)	28,778	31.2%	26,776	32.3%	2002	21.1%	< 0.001
BMI > 40 (kg/m^2^) (%)	3115	3.4%	2871	3.5%	244	2.6%	< 0.001

*SD* standard deviation, *BMI* body mass index, *SBP* systolic blood pressure, *DBP* diastolic blood pressure, *HR* heart rate, *TC* total cholesterol, *LDL-C* low density lipoprotein cholesterol, *HDL-C* high density lipoprotein cholesterol, *TG* triglycerides, *DM* diabetes mellitus, *AH* arterial hypertension, *MS* metabolic syndrome, *RF* risk factors, *CHD* coronary heart disease

### Cardiovascular risk assessment in middle-aged men with and without dyslipidemia

**Fig. 1:**
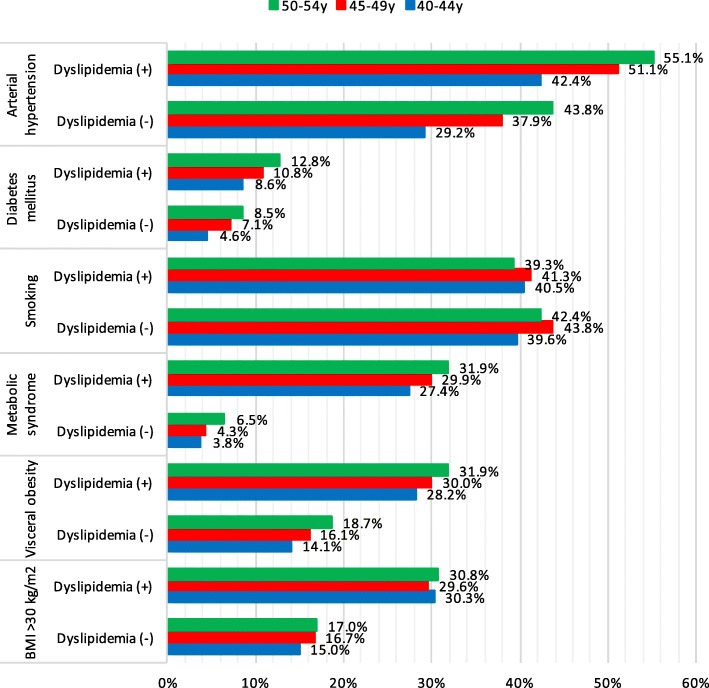
Prevalence of different cardiovascular risk factors in men of different age with and without dyslipidemia

This study included 38,412 men aged 40–54: 33403 (87.0%) with dyslipidemia and 5009 (13.0%) without dyslipidemia. Mean value of TC in men was 6.07 ± 1.10 mmol/l, LDL-C – 3.92 ± 0.98 mmol/l, HDL-C – 1.39 ± 0.47 mmol/l and TG – 1.86 ± 1.45 mmol/l. Prevalence of different cardiovascular risk factors in dyslipidemic and non-dyslipidemic men is presented in Fig. 1. Men with dyslipidemia had all main risk factors, except smoking, significantly more often compared to males without dyslipidemia: AH (49.6% vs. 36.6%, *p* < 0.001), DM (10.8% vs. 6.6%, *p* < 0.001), abdominal obesity (30.1% vs. 16.2%, *p* < 0.001), MS (29.8% vs. 4.8%, *p* < 0.001) and obesity (30.3% vs. 16.1%, *p* < 0.001). The prevalence of DM, AH, abdominal obesity, MS and obesity increased with age in both dyslipidemic and non-dyslipidemic men groups. Males with dyslipidemia had higher prevalence of DM, AH, abdominal obesity, MS and obesity in all age groups in comparison with control group (Fig. 1). Smoking was more prevalent in men aged 40–54 without dyslipidemia compared to group with dyslipidemia (41.8% vs. 40.3%, *p* < 0.001). The highest frequency of smoking in males without dyslipidemia was observed in 45–49-year-olds (43.8%) followed by 50-55y (42.4%) and 40-44y (39.6%) groups. Prevalence of smoking was lower in men older than 50 years compared to younger males in both dyslipidemic and non-dyslipidemic groups (Fig. 1). Also, men with dyslipidemia tended to have family history of CHD, unbalanced diet and insufficient physical activity more often compared to men without dyslipidemia (23.9% vs. 20.6%, *p* < 0.001; 65.5% vs. 54.1%, *p* < 0.001; 47.3% vs. 39.5%, *p* < 0.001, respectively).

### Cardiovascular risk assessment in middle-aged women with and without dyslipidemia

**Fig. 2:**
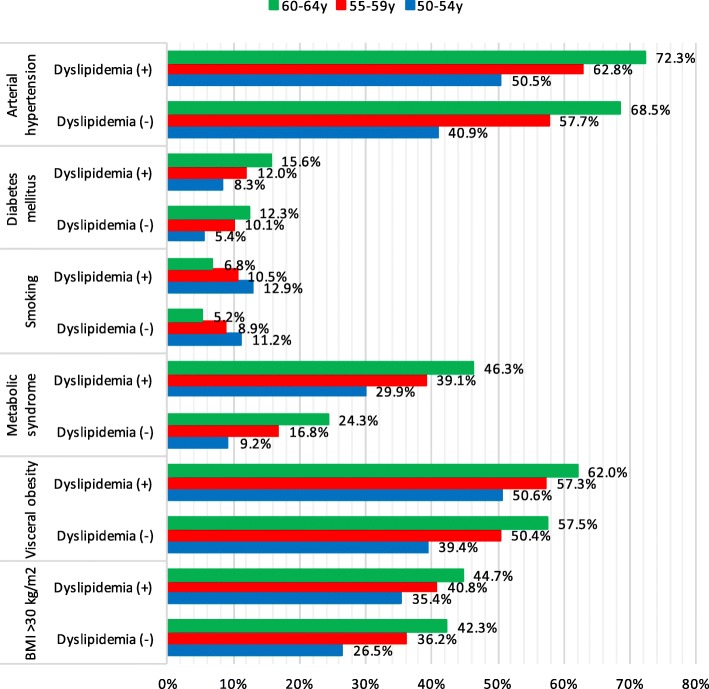
Prevalence of different cardiovascular risk factors in women of different age with and without dyslipidemia

This study included 53,961 women aged 50–64: 49490 (91.7%) with dyslipidemia and 4471 (8.3%) without dyslipidemia. Mean laboratory values of women were: TC – 6.41 ± 1.11 mmol/l, LDL-C – 4.12 ± 1.01 mmol/l HDL-C – 1.63 ± 0.45 mmol/l and TG – 1.53 ± 0.96 mmol/l. Prevalence of different cardiovascular risk factors in dyslipidemic and non-dyslipidemic women of different age is shown on Fig. 2. Women aged 50–64 with dyslipidemia had all main cardiovascular risk factors significantly more frequently compared to females without dyslipidemia (DM (11.3% vs. 8.0%, *p* < 0.001), AH (59.9% vs. 50.7%, *p* < 0.001), abdominal obesity (55.6% vs. 45.8%, *p* < 0.001), smoking (10.6% vs. 9.4%, *p* < 0.001), MS (36.9% vs. 14.2%, *p* < 0.05) and obesity (39.5% vs. 32.1%, *p* < 0.001)). The prevalence of DM, AH, abdominal obesity, MS and obesity increased with age in both dyslipidemic and non-dyslipidemic groups. Subjects with dyslipidemia had higher prevalence of DM, AH, abdominal obesity, MS and obesity in all age groups in comparison with control group (Fig. 2). Women with dyslipidemia reported smoking more often in all age groups compared to women without dyslipidemia (*p* < 0.001). Frequency of smoking was lower in older women in both dyslipidemic and non-dyslipidemic groups compared to younger females (Fig. 2).

### Dyslipidemia and concomitant risk factors

**Fig. 3:**
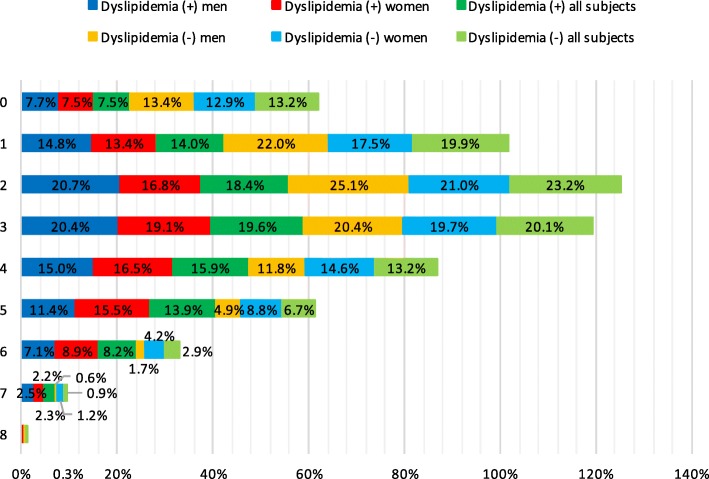
Distribution of the number of cardiovascular risk factors in study population

Adults with dyslipidemia more often had ≥3 concomitant risk factors in comparison with control group (60.1% vs. 43.8%, *p* < 0.001). Across the study population adults without dyslipidemia tended to have less risk factors while women and men with dyslipidemia had bigger number of cardiovascular risk factors (Fig. 3). 7.5% of dyslipidemic and 13.2% of non-dyslipidemic patients did not have any other conventional risk factors. The distribution of the number of cardiovascular risk factors in study cohort is presented in Fig. 3.

## Discussion

Numerous studies have established dyslipidemia [18], arterial hypertension [19], smoking [20] and diabetes mellitus [21] as main independent cardiovascular risk factors. Modifying them is proven to be the key approach to lower cardiovascular mortality [7, 22, 23]. More than 90% of acute coronary events occur in individuals with at least one expressed risk factor [24]. Dyslipidemia was responsible for 15.1% of CVD death risk in the EURIKA population [25]. The prevalence of dyslipidemia differs widely according to social, economic, ethnic and cultural aspects of specific population groups. It varies between 34.0–41.9% in China [26, 27], an estimated 53.0% of adults in the USA [28], 76.4% in German adults [29] and 79.0% in India [30]. According to previous studies in Lithuania, hypercholesterolemia was present in 51.3% women and 52.2% men aged 25–64 years (CINDI) and 81% men and 87% women aged 35–64 in MONICA study [31, 32]. In our study, prevalence of dyslipidemia in middle-aged Lithuanians was 89.5% in 2016, slightly increasing from 2009 (89.1%) as reported by LitHiR programme. This data was consistent with the results of EURIKA study where prevalence of dyslipidemia was 89.4% in primary prevention centers across Europe [25].

Cigarette smoking is one of the most important modifiable cardiovascular risk factors as it accounts for about 36% of the population attributable risk of acute myocardial infarction [33]. Smoking is related to alterations in serum lipid profile, especially with reduction low HDL-C [34, 35]. Some of the previous studies reported that smoking causes an increase in TC, LDL-C, triglycerides, while other studies have presented results of smoking decreasing levels of TC and LDL-C [36, 37]. Possible reasons for these inconsistencies of studies’ results could be ethnic differences, age, gender, different diet, lifestyle and other factors [38]. Our study found the trend of higher prevalence of smoking in non-dyslipidemic men gender-specific, as men without dyslipidemia smoked significantly more often compared to men with dyslipidemia, while women with dyslipidemia smoked more frequently compared to non-dyslipidemic females. Some of researchers have suggested that association of smoking with the risk of dyslipidemia is stronger in women than in men and women who smoke could be more susceptible to develop dyslipidemia than men smokers [34].

The prevalence of obesity, MS and type 2 diabetes is rising at alarming rates and increasing the cost of obesity-related medical care [39]. Research referring to association between obesity and dyslipidemia is not definite: some studies found that dyslipidemia correlated with BMI in men and waist circumference in women [40] while others described associations between waist circumference and dyslipidemia in both males and females whereas relationship between BMI and abnormal lipid levels was significant only in women [41, 42]. Our study showed that subjects with dyslipidemia tended to have MS as well as abdominal obesity significantly more often in comparison with control group. With increasing overweight and obesity class, there is an increase in the frequency of dyslipidemia: Nguyen et al. found that prevalence of dyslipidemia was 8.9% in normal weight (BMI < 25) adults and 19% in obesity class III (BMI ≥40) with highest prevalence of dyslipidemia in obesity class II (BMI 35–39.9) (20.6%) [43]. In our study population, adults with dyslipidemia most often were overweight (38.9%), followed by obesity class I-II (32.3%), subjects with normal weight (25.4%) and obesity class III (3.5%). Diabetes is associated with atherogenic dyslipidemia, which could be one of the major determinants of high cardiovascular risk in patients with DM [44]. In this study, adults with dyslipidemia had DM more often compared to non-dyslipidemic subjects. Therefore, managing diabetic dyslipidemia requires an integrated approach: lifestyle changes and combined pharmacological interventions are the most important treatment strategies [45].

Dyslipidemia and hypertension are the two most prevalent as well as most commonly co-existing major cardiovascular risk factors [9, 46]. Abnormal lipid levels are one of the earliest metabolic disturbances in adults with hypertension, occurring in more than one-third of hypertensive patients [46]. Dyslipidemia increases cardiovascular risk more than twice for subjects with hypertension confirming that risk factors interact with each other [47, 48]. According to previous studies analyzing LitHiR data, dyslipidemia was present in 91.5% of patients with AH [14]. In our study, prevalence of hypertension in the whole study population was 54.5% (55.8% of dyslipidemic patients). Prevalence of dyslipidemia in Lithuania (89.7%) is estimated to be higher than prevalence of hypertension while AH remains the most common risk factor for both developed and still developing countries worldwide [49, 50].

The impact of individual risk factors is well established but evaluation of cardiovascular risk requires assessment of multiple risk factors as clustering of CVD risk factors leads to higher risk of developing cardiovascular events by having synergistic, rather than additive, effects on total risk [3, 51, 52]. According to study by Wu et al. percentage of subjects with two or more cardiovascular risk factors varied from 36.0% in young men to 67.4% in males aged 50–59 and from 6.5% in young women to 50.3% of older (50-59y) females [53]. Frequency of multiple risk factors increased with age in both men and women [53]. According to our data, dyslipidemia was often accompanied by other cardiovascular risk factors – almost two thirds of middle-aged subjects with dyslipidemia had three or more cardiovascular risk factors and only 7.5% had no other risk factors. While typical high-risk profile of middle-aged Lithuanian has been described as having dyslipidemia, hypertension and abdominal obesity [54], it is clear that high cardiovascular risk in Lithuania is impacted by combination of these different risk factors. Studies have shown that greater number of risk factors is related to correspondingly poorer clinical outcome [55]. Based on these revelations, it is clear that optimal therapeutic CVD prevention strategy requires targeting 2 or more of risk factors collectively [46].

Despite very high prevalence of dyslipidemia, frequent coexistence of several cardiovascular risk factors and high cardiovascular mortality in Lithuania, the average estimated 10-year risk of fatal CVD disease based on the SCORE equation in our study patients was moderate (1.87%). This finding was not compatible with the EURIKA study results where average 10-year risk of CVD death was 8.2% across Europe [25]. This revelation may propose that high-risk SCORE equation could underestimate the risk in Lithuania and other Eastern European countries with extremely high rates of CVD mortality despite paper by Vikhireva et al. showing that high-risk SCORE was a significant risk predictor in Lithuania and other Central and Eastern European countries [56]. Other possible explanation could be the SCORE index underestimating risk in patients with certain risk factors not included in the equation, such as sedentary lifestyle, visceral obesity, a family history of premature CVD, or presence of subclinical atherosclerosis [3, 25]. This discovery could be the basis of future studies examining potential reasons for the disparity between calculated risk by SCORE equation and actual risk in our country.

To overcome the existing situation, it is essential to improve the control of multiple risk factors, which have an important role in cardiovascular pathology. There is good quality evidence of early and integrated approach to correction of dyslipidemia and we need to raise awareness about the benefit of timely detection and treatment of abnormal lipid levels.

## Limitations of the study

The present study examined a sample of men aged 40–54 years and women aged 50–64 years. A future study is needed to examine the younger and the older samples. Some risk factors, which are important in risk assessment, such as psychosocial factors, social class and others, were not taken into account. Also, dyslipidemia as defined in this study, cover broad spectrum of lipid abnormalities, so very high prevalence of this disorder was detected. Further studies are needed to evaluate prevalence of different types of dyslipidemia in this population.

## Conclusions

Dyslipidemia is very common in middle-aged Lithuanians without overt cardiovascular disease. Although prevalence of cardiovascular risk factors increases with age, dyslipidemia is associated with greater probability of having diabetes mellitus, arterial hypertension, abdominal obesity, metabolic syndrome and obesity, except for smoking, in all age groups compared to adults without dyslipidemia. Men and women with dyslipidemia have a higher total number of cardiovascular risk factors which synergistically increases absolute risk of CVD. The average 10-year risk of CVD death in patients with dyslipidemia was 1.95%. Our observations emphasize the importance of properly diagnosing and treating dyslipidemia as well as other concomitant risk factors in order to reduce burden of cardiovascular disease in Lithuania.
